# Case Report: A man with septic shock developed convulsions

**DOI:** 10.3389/fmed.2025.1605482

**Published:** 2025-08-08

**Authors:** Linxiang Huang, Peiliang Gao, Xueying Fu, Yuran Wang, Jihong Zhu, Guiying Dong

**Affiliations:** ^1^Peking University Hepatology Institute, Peking University People’s Hospital, Beijing, China; ^2^Emergency Department, Peking University People’s Hospital, Beijing, China

**Keywords:** septic shock, convulsion, case report, infective endocarditis, erysipelas, deep venous thrombosis

## Abstract

Embolism has variable manifestations in infective endocarditis (IE) and might be misinterpreted in clinical settings. In this study, we reported the case of a 67-year-old man with erysipelas and septic shock who had developed a new onset of blurred vision and subsequently convulsions and multiple cerebral infarctions. Valve vegetations and patent foramen ovale were negative on the first echocardiography. After the neurologic symptom occurred, repeated echocardiography was performed and revealed vegetations on the mitral valve. As the patient had immunological complications, we suggested prolonged antibiotic therapy, but the patient requested an early discharge due to financial concerns. Under such circumstances, administering a long-acting penicillin G via an intramuscular injection once was an alternative therapy before discharge. For patients with bloodstream infection who develop any new onset symptoms related to arterial occlusion, infective endocarditis should be suspected, and repeated echocardiography is necessary.

## Introduction

Acute infective endocarditis has a short onset and the progression worsens rapidly, manifesting as high fever, sepsis, and other systemic symptoms. A new-onset heart murmur is a distinguishable sign in the diagnosis of infective endocarditis. However, it has been reported that new murmurs are present in less than half of the cases (1). Arterial embolism has variable non-specific manifestations depending on the various affected organs involved in infective endocarditis and could even be the first presentation. Therefore, early recognition of these signs is essential in diagnosing patients with fever and sepsis. The microbiological profile of infective endocarditis has also changed, with staphylococci overtaking streptococci as the most common cause of the disease (2). In this study, we report the case of 67-year-old man presented with fever and sepsis induced by erysipelas in the lower limbs, with complaints of significant fatigue and blurred vision before admission, and subsequently developed multiple cerebral infarctions. Infective endocarditis was diagnosed following repeated echocardiography. Therefore, clinicians should raise awareness of repeated echocardiography when results are negative during the first examination and when infective endocarditis is strongly suspected (3). Blurred vision is a possible indication of arterial embolism and should be taken seriously (4).

## Case presentation

A 67-year-old man presented to the emergency department with a 10-day history of fever (fluctuating at 38.5°C), fatigue, muscle pain, cough with yellow sputum, shortness of breath, and tenderness in the lower limbs. His blood pressure was 65/41 mmHg when measured at home, and he was admitted to the local hospital 9 days ago, where he was diagnosed with septic shock and received treatment including rehydration, vasopressors, and antibiotics. After a few days of treatment, he began to suffer from significant fatigue and blurred vision. He had a history of anti-neutrophil cytoplasmic antibody (ANCA)-associated vasculitis with focal proliferative glomerulonephritis 10 years ago (creatinine ranging from 160 to 180 umol/L until present) and hypertension.

**Figure 1:**
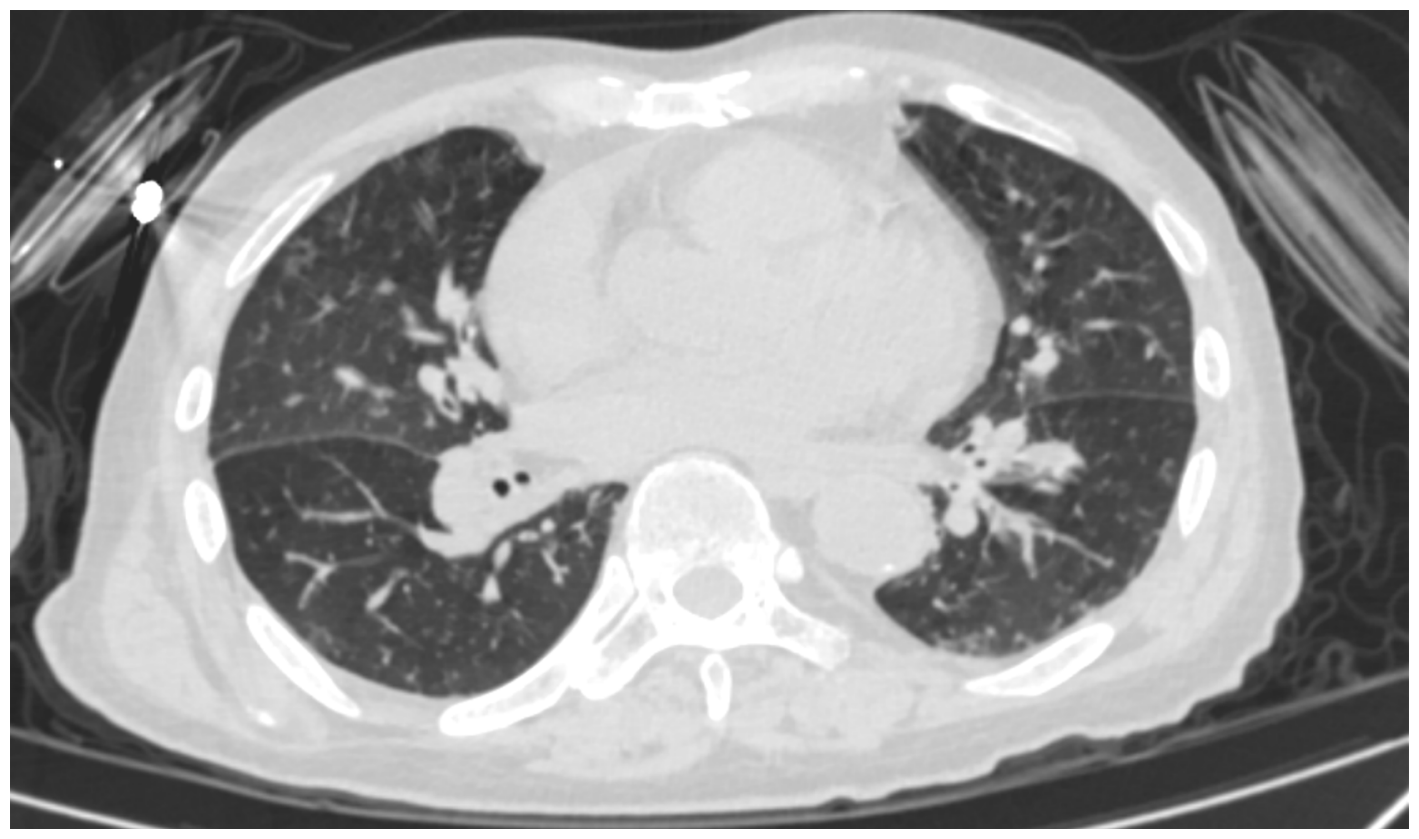
Chest CT of the patient.

**Figure 2:**
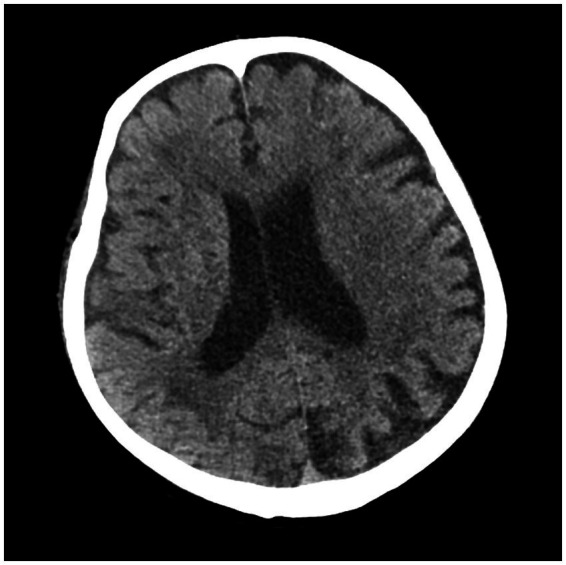
Cranial CT of the patient.

Upon examination, he was alert, with a temperature of 38.5°C, a pulse rate of 102 beats/min, a blood pressure of 82/54 mmHg, a respiratory rate of 20 beats/min, and an oxygen saturation of 98% in room air. He had no rhonchi or crackles on pulmonary auscultation, and no heart murmurs were detected. He had redness, swelling, and tenderness in his lower limbs, most predominantly on the left one. Table 1 shows the results of the relevant blood tests. Chest (Figure 1) and cranial (Figure 2) computed tomography (CT) were performed. Vascular ultrasonography of the lower limbs detected thrombosis in the great saphenous vein, popliteal vein, and posterior tibial artery of the left and in the distal femoral vein, superficial femoral vein, popliteal vein, and fibular vein of the right. Echocardiography revealed mild regurgitation of the mitral and tricuspid valves. Later, septic shock with pneumonia and erysipelas was diagnosed.

**Table 1:** Relevant blood tests.

Test	Result	Normal range
White cell count	46.53 × 10⁹/L	3.5–9.5 × 10⁹/L
Neutrophils	45.10 × 10⁹/L	1.8–6.3 × 10⁹/L
C-reactive protein	236.3 mg/L	<10 mg/L
Procalcitonin	>100.000 ng/mL	<0.500 ng/mL
Creatinine	229 umol/L	59–104 umol/L
D-dimer	2,625 ng/mL	<243 ng/mL

**Figure 3:**
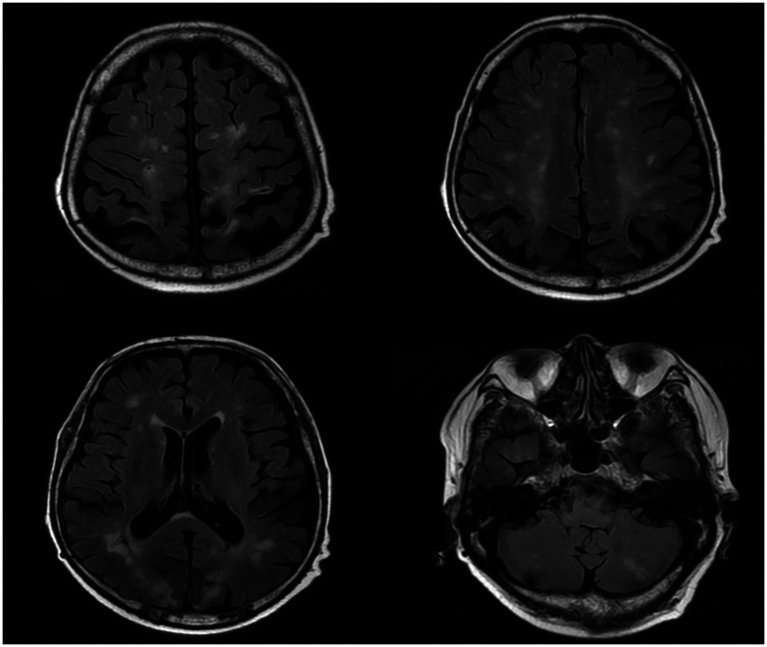
Repeated cranial CT of the patient.

**Figure 4:**
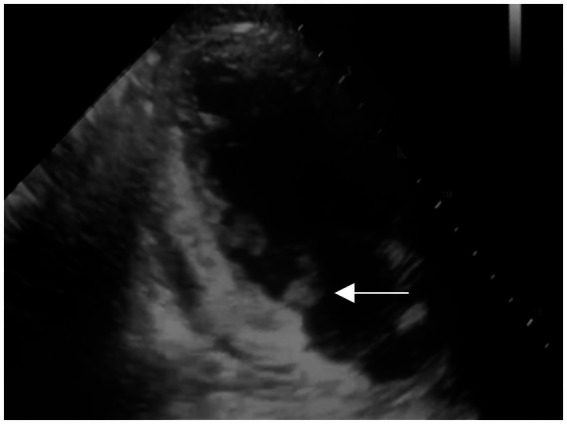
Transthoracic and contrast echocardiography of the patient 7 days after the first echocardiography (white arrow: mitral valve vegetation).

After admission, he received treatment, including rehydration, vasopressor (metaraminol bitartrate), antibiotics (imipenem-cilastatin sodium and moxifloxacin), prednisone (5 mg qd), and other support treatment. Pathogens were negative after 7 days of blood culture. On day 7 of treatment, the patient had a sudden convulsion with repetitive jerks, loss of consciousness, and eyes gazing to the right. A measure of 3 mg midazolam was injected to control symptoms, and cranial CT (Figure 3) was performed again, which revealed multiple low-density regions of both the cerebral and cerebellar hemispheres. Patent foramen ovale (PFO) was negative on transthoracic and contrast echocardiography, but it showed 0.7 cm*0.6 cm vegetation on the mitral valves (Figure 4, white arrow). Up to this point, infectious endocarditis was diagnosed.

As the blood culture was negative, penicillin was the first choice of empirical therapy for IE (5). Piperacillin-tazobactam was used intravenously. After 7 days of treatment, the patient’s WBC decreased to 3.33 × 10^9^/L and procalcitonin (PCT) levels decreased to 0.331 ng/mL. We recommended a prolonged course of antibiotics (4–6 weeks) to reduce the recurrence rate, but the patient’s family requested an early discharge due to financial burden. After consultation with our infectious disease department, 1.2 million units of benzathine benzylpenicillin—a long-acting penicillin G—via an intramuscular injection once was administered as an alternative therapy before the patient’s discharge. He was discharged with an activity of daily living (ADL) score of 45/100, compared to 20/100 on admission.

Due to financial and other concerns, the patient did not return to our hospital for follow-up visits. One year after discharge, we conducted a telephone follow-up. The patient had not undergone repeat echocardiography or cranial CT/MRI since discharge. He continued to experience intermittent seizures and reported a decline in cognitive function. He had once been admitted to the emergency department of a local hospital for seizure that required intubation. At the time of follow-up, his vital signs were stable, and he had no recurrent fever; the previous episode of erysipelas had resolved. The patient was taking oral valproate and prednisone to manage his underlying condition.

## Discussion

This patient presented with septic shock and tenderness of the lower limbs during the first consultation. IE is an important differential diagnosis; however, no significant findings were reported on the first echocardiography. After the sudden convulsion, mitral valve vegetation was detected by repeated echocardiography (7 days after the initial transthoracic echocardiography), with IE being a suspected cause of cerebral infarctions. Bacteremia is essential in the diagnosis of IE (2), and in this case, pneumonia and erysipelas are both important factors for disease progression. However, due to the early use of antibiotics before admission, the blood culture was negative. However, with one major criterion and three minor criteria met according to the modified Duke criteria (6), the diagnosis of IE remained valid. The complaint of blurred vision might have been an early signal of embolism shedding from mitral valve vegetation.

In patients who present with multifocal neurological manifestations, PFO, IE, thrombophilia, immunothrombosis, and sepsis-associated encephalopathy (SAE) should be suspected. For patients with bloodstream infection who have any new-onset symptoms related to artery occlusion, infective endocarditis should be suspected. However, the negative results on the first echocardiography made it challenging to diagnose IE. Besides, these patients with deep venous thrombosis who had cerebral infarctions, the first differential diagnosis is usually patent foramen ovale. The sensitivity of transthoracic echocardiography in the diagnosis of PFO was reported to be 79–94% (7). In this case, PFO was negative, and other differential diagnoses were considered.

SAE refers to a diffuse brain disorder that occurs secondary to infection within the body, excluding overt central nervous system (CNS) infection. It is commonly observed in critically ill patients, and the severity of SAE can vary from mild delirium to deep coma. Seizures and myoclonus are rare, and the cranial nerves are almost always spared (8). These features were largely inconsistent with this patient’s presentation, and therefore, SAE was ruled out.

The recommended treatment for IE can be divided into two phases. In the early phase, patients with IE should receive surgical or other appropriate interventions when receiving intravenous antibiotics for approximately 10 days, or until blood cultures become negative. The early phase is followed by the continuation phase, during which patients with complex infections should complete a full 4–6 weeks course of antibiotics administered either orally or intravenously. Following antibiotic therapy, close post-discharge monitoring are crucial (9). However, the patient’s family ultimately declined to receive further treatment and requested to be discharged. A long-acting penicillin G was chosen as an alternative therapy for administration before the patient’s discharge.

It has been reported that ANCA was found in a substantial proportion (18–33%) of patients with IE. ANCA-positive patients with infective endocarditis were reported to have a longer disease duration, more frequent purpura, and kidney involvement compared to patients with ANCA-negative patients (10). In this case, the patient had a history of ANCA-associated vasculitis and renal injury, which may have increased the risk of disease development.

In acute ischemic stroke patients with IE, high rates of intracerebral hemorrhage and low rates of favorable outcome were reported after intravenous thrombolysis (11). Therefore, it is generally believed that patients with cerebral embolism caused by IE should not receive thrombolytic therapy.

## Conclusion

Embolism exhibits variable manifestations in infective endocarditis and may be misinterpreted in clinical settings. For patients with bloodstream infections who develop any symptoms related to arterial occlusion, clinicians should be vigilant for infective endocarditis, and repeated echocardiography is recommended. A full course of antibiotic therapy and close post-discharge monitoring are crucial.

## Data Availability

The original contributions presented in the study are included in the article/supplementary material, further inquiries can be directed to the corresponding author.
